# Analysis of Families of Curves

**DOI:** 10.6028/jres.067A.027

**Published:** 1963-06-01

**Authors:** John Mandel, Frank L. McCrackin

## Abstract

A systematic approach is presented for fitting empirical expressions to data depending on two variables. The problem can also be described as the simultaneous fitting of a family of curves depending on a parameter.

The proposed method reduces a surface fitting problem to that of fitting a few functions of one variable each. First, the surface is expressed in terms of these one-variable functions, and using an extension of two-way analysis of variance, the accuracy of this fit is assessed without having to determine, at this point, the nature of the one-variable functions. Then, the one-variable functions are fitted by customary curve-fitting procedures.

For illustration, the method is applied to two sets of experimental data.

## 1. Introduction

A frequently occurring situation in scientific work is one in which the relationship between two quantities is examined for a series of values of a third quantity. For example, in the thermodynamic studies of gases the pressure-volume relationship may be examined at various temperatures. The results of such experiments are often presented in terms of a one-parameter *family* of curves. Alternatively, one may describe the problem as the fitting of a surface in a space of three dimensions.

An analysis of a set of data (or curves) of this type follows one of two possible lines: either a model is postulated on the basis of physicochemical hypotheses, in which case the main purpose of the analysis is to verify the adequacy of this model, and possibly to estimate certain constants occurring in the model; or there exists no pertinent theory, in which case the problem consists in finding a satisfactory empirical representation of the data. Thus, in our example, one might postulate Van der Waals equation:

$$\left(p+\frac{a}{V^{2}}\right)\left(V-b\right)=RT$$ (1)

where *p, V*, and *T* represent pressure, volume, and temperature, *R*, the gas constant, and *a* and *b* two constants to be inferred from the data. The postulation of this equation would put the problem in the first category. On the other hand, the experimenter may desire to determine the form of the equation that best represents his data, without committing himself to any specific preconceived equation such as (1). In that case, which constitutes a problem of the second category, the choice of a suitable equation may present considerable difficulties. There exist few, if any, guidelines to assist one in the selection, and trial and error is the only way by which a particular equation is finally chosen. A widely used statistical procedure for fitting curves and surfaces is the method of least squares. Application of this method requires that some specific functional form be agreed upon *prior* to the fitting process. This process serves to estimate the unknown parameters and to evaluate the adequacy of the fit in terms of the smallness of the residuals. There is no assurance, by this method, that a much better fit might not be achieved by an entirely different functional form. Also, if the fit turns out to be inadequate, the method of least squares yields little, if any, information regarding the direction in which one ought to search for a more appropriate model.

In this paper, the empirical fitting of a family of curves is attacked in a systematic way. Mathematical expressions are used involving functions that depend each on one variable only. The nature of each of these functions is left entirely open in the initial fitting process, and the adequacy of the fit is judged without having to specify the nature of these functions. Thus, one need not estimate the values of any parameters before judging the success of the fit.

The specific examples presented in this paper are used only to illustrate the mathematical approach and not to propose alternative equation of state, either for rubber or for ethylene.

## 2. Generalized Model

For the sake of clarity, we shall discuss the problem first in terms of eq (1). Rewriting eq (1) as:

$$p=\left(\frac{-a}{V^{2}}\right)+\left(\frac{R}{V-b}\right)T$$ (2)

we see that for any particular value of *V*, it represents simply a linear relationship between *p* and *T.* Thus, for any value of *V*, a plot can be made of *p* versus *T*, and a straight line fitted to the plotted points. If data are available for different values of *V*, this method will result in a collection of straight lines, one for each value of *V*. The slope of the straight line, corresponding to any given value of *V*, is $\frac{R}{V-b}$ and the intercept is $\frac{-a}{V^{2}}$. Thus, by studying the relationship between the experimentally determined slopes and the corresponding values of *V*, one can obtain an estimate of the parameter *b.* Similarly, from the intercepts an estimate of *a* can be obtained.

So far, no new technique of analysis has been introduced, and the procedure is entirely contingent on the linearity of *p* in terms of *T.* Note, however, that in fitting each straight line, no use has been made of the fact that the slope depends on *V* in accordance with the function $\frac{R}{V-b}$ or that the intercept is inversely proportional to *V^2^*. It is only in the estimation of *b* and *a* that consideration has been given to these facts.

Suppose, now, that the experimenter is not committed to eq (2) as the only possible representation of his data, or that, in fact, he knows this equation to be unsatisfactory for that purpose. It is then possible to suggest an immediate generalization of eq (2), far less restrictive than this equation, that may be more adequate as a representation of the data.

We note that eq (2) belongs to the general class.

p=f(V)+g(V)h(T) (3)

where *f* and *g* are two distinct functions of volume only, while *h* is a function of temperature only. Equation (3) is more general than eq (2) in that no assumptions are made regarding the form of the functions *f*, *g*, and *h.* For example, *h*(*T*) may be a quadratic, or an exponential, or any other desired function of *T.* Nor is it necessary to assume that *f*(*V*) and *g*(*V*) obey the functional forms $\frac{-a}{V^{2}}$ and $\frac{R}{V-b}$ required by Van der Waal’s equation. Any dependence of *h* on *T* and of *f* and *g* on *V* is admissible in the general formulation of eq (3).

We will adopt as our generalized model that represented by eq (3). First we describe a method for fitting the model represented by eq (3) and for evaluating the adequacy of the fit. Then we illustrate the usefulness of this model by applying it to two sets of experimental data.

## 3. Analysis of the Generalized Model

Let the data be in the form of a rectangular array, in which each row is associated with a particular value of *V*, and each column with a particular value of *T.* Each cell of the array then contains the value of pressure corresponding to the volume and temperature values represented by the row and column intersecting in that cell. Such an arrangement is shown in table 1.

The main difficulty in fitting eq (3) lies in our ignorance of the function *h*(*T*). Indeed, eq (3) expresses for any given value of *V*, a linear relation between *p* and *h*(*T*). If *h*(*T*) is known for each *T*, the straight line corresponding to each value of *V* can at once be plotted and the nature of the functions *f*(*V*) and *g*(*V*) can then be determined by studying the slopes and intercepts of the lines as functions of *V.* Let us note, however, that a similar analysis can be made as soon as we have a set of values linearly related to *h*(*T*). For if a function *H*(*T*) is defined by

H(T)=α+βh(T) (4)

eq (3) can be written

p=A(V)+B(V)H(T) (5)

with

$$A\left(V\right)=f\left(V\right)-\frac{\alpha }{\beta }g\left(V\right)$$ (6a)

$$B\left(V\right)=\frac{g\left(V\right)}{\beta }$$ (6b)

Then eq (5) also represents, as does eq (3), a linear relationship between *p* and *H*(*T*) for each value of *V*. If *H*(*T*) is known, the functions *A*(*V*) and *B*(*V*) may then be determined from the linear fits of *p* versus *H*(*T*), for different values of *V*. Now when *h*(*T*) is unknown, there exist nevertheless many functions *H*(*T*) the values of which can be inferred from the data for all *T* values represented in the table. One of these functions is given by the column averages ${\bar{p}}_{T}$ of table 1. This follows at once by averaging both members of eq (3) over all rows, for any given value of *T:*

$${\bar{p}}_{T}=\bar{f}+\bar{g}h\left(T\right).$$ (7)

This function belongs indeed to the class of *H*(*T*) defined by eq (4). For reasons of statistical convenience, a preferable choice is given by

$$C_{T}={\bar{p}}_{T}-\bar{\bar{p}}$$ (8)

where $\bar{\bar{p}}$ is the grand average of all ${\bar{p}}_{T}$ values in the table. When *H*(*T*) is selected to be *C_T_*, as defined by eq (8), we will refer to the corresponding representation by eq (5) as the “standard form.” Thus, the standard form is given by:

$$p=A_{V}+B_{V}C_{T}$$ (9)

where *C_T_* is defined by (8) and:

$$A_{V}=f\left(V\right)-\frac{\bar{f}-\bar{\bar{p}}}{\bar{g}}g\left(V\right)$$ (10a)

$$B_{V}\frac{g\left(V\right)}{\bar{g}}$$ (10b)

From (10b) it follows that the average of *B_V_* over all rows is equal to unity. On the other hand, eq (8) shows that the average of the *C_T_* over all columns is equal to zero. Thus:

$$\bar{B}=1\;\text{and}\;\bar{C}=0.$$ (11)

It is easily verified that these two conditions are necessary and sufficient for assuring that the representation is in the standard form. Therefore, a function of two variables, as represented in table 1, may be approximated, in the form of eq (9), by three single—variable functions. The function *C_T_* of temperature is first computed from the column averages of table 1 by eq (8). A linear fit of each row of the table versus *C_T_* then gives the values of the functions *A_V_* and *B_V_* as the intercepts and the slopes of the fitted lines.

An analytical formula for the function of two variables may now be obtained by fitting empirical formulas to the curves *C_T_* versus *T, A_V_* versus *V*, and *B_V_* versus *V.*

## 4. Statistical Model

So far we have not considered errors of measurement. Let us now assume that the experiment has been conducted in such a way that *V* and *T* are controlled and *p* is a measurement subject to experimental error. Then eq (3) becomes:

$$p=f\left(V\right)+g\left(V\right)h\left(T\right)+\epsilon^{\prime }$$ (3a)

where *ϵ*′ is a random error of zero expectation. For greater generality, the first member in eq (3a) can be replaced by any suitable function of *p.* In work dealing with equations of state, such as pressure-volume-temperature relationships, it is customary to study the quantity *pV.* Replacing *p* by *pV* in the left-hand side of (3a) would visibly not change the functional nature of the right-hand side of this relation and it would generally result in greater homogeneity in the variance of the error term. Representing the measured quantity, or any appropriate function of it (as in this case *pV*) by *Z_V,T_*, we have the general relation

$$Z_{V,T}=f\left(V\right)+g\left(V\right)h\left(T\right)+\epsilon$$ (12)

which can be written in the standard form:

$$Z_{V,T}=A_{V}+B_{V}C_{T}+\epsilon$$ (13)

where $\bar{B}=1$ and $\bar{C}=0$. Specifically, *C_T_* is defined by

$$C_{T}={\bar{Z}}_{T}-\bar{\bar{Z}}$$ (14)

where ${\bar{Z}}_{T}$ is the column average for column *T* and $\bar{\bar{Z}}$ the grand average in table 1, the cell entries of which are *Z_V, T_.* In regard to the errors, *ϵ*, we will assume that they are normally and independently distributed constituting a sample from a normal population of zero mean, and variance equal to *σ*^2^.

Under these assumptions, the values *Z_V, T_* and ${\bar{Z}}_{T}$ (from which the *C_T_* are calculated) are no longer statistically independent, nor are ${\bar{Z}}_{T}$ and *C_T_* independent. It has, however, been shown [1]^1^ that the following analysis is not invalidated by this circumstance.

## 5. Statistical Analysis

Denote by *m* the number of rows of table 1, and by *n* the number of its columns. For each row, a straight line is fitted to the set of points (Z, *C*) using the usual method of linear regression. This yields the estimates,

$${\hat{A}}_{V}=\frac{\sum_{T}Z_{V,T}}{n}$$ (15)

$${\hat{B}}_{V}=\frac{\sum_{T}Z_{V,T}C_{T}}{\sum_{T}C_{T}^{2}}$$ (16)

and an estimate of the variance about the regression line:

$${\hat{V}}_{\left(\epsilon \right)}=\frac{\sum_{T}{\left[Z_{V,T}-\left({\hat{A}}_{V}+{\hat{B}}_{V}C_{T}\right)\right]}^{2}}{n-2}.$$ (17)

Since the variance of *ϵ* is assumed to be the same for all values of *V*, the *m* estimates given by eq (17) for the *m* values of *V* may be pooled. How this is to be done will be shown in the discussion on the analysis of variance. Note, however, that an inspection of the *m* values of ${\hat{V}}_{\left(\epsilon \right)}$ is of considerable interest, especially for the detection of trends related to the magnitude of *V.* A pooled value is meaningful only in the absence of such trends.

From (17), or from a pooled value of ${\hat{V}}_{\left(\epsilon \right)}$, estimates of the standard errors of ${\hat{A}}_{V}$ and ${\hat{B}}_{V}$ may be obtained by the usual formulas.

## 6. Case of Concurrence

Among the many possibilities for the structure of a family of curves, two special cases deserve particular attention. The first concerns a family of “parallel” curves. In this case, the straight lines resulting from the application of the method described in this paper will also be parallel. Their slopes are then independent of *V* and all equal to unity so that the model reduces to the “additive” type.

$$Z_{V,T}=A_{V}+C_{T}+\epsilon =A_{V}+{\bar{Z}}_{T}-\bar{\bar{Z}}+\epsilon .$$ (18)

The second special case is that in which all the curves of the family pass through a common point. We denote this situation as the “concurrent” case. When the curves are concurrent, then so are the straight lines resulting from our analysis. Now a necessary and sufficient condition for a collection of straight lines of the type

Z=f(V)+g(V)h(T) (19)

to concur, is that a linear relation exist between *f*(*V*) and *g*(*V*). For if *h*(*T*_0_), *Z*_0_ are the coordinates of the common point, the following identity must hold for all *V;*

$$Z_{0}=f\left(V\right)+g\left(V\right)h\left(T_{0}\right)$$

or

$$f\left(V\right)=Z_{0}-\left[h\left(T_{0}\right)\right]g\left(V\right).$$ (20)

This equation expresses a linear relation between *f*(*V*) and *g*(*V*), since *Z*_0_ and *h*(*T*_0_) are numerical constants. Conversely, if this linear relation holds, then the entire set of straight lines passes through the point [*h*(*T*_0_), *Z*_0_], and hence is concurrent.

The importance of the concurrent model is that in it, the algebraic expression of the structure of the family of curves becomes quite simple. Indeed, replacing in eq (19), the quantity *f*(*V*) by its expression given by eq (20), we obtain

$$Z=\left[Z_{0}-h\left(T_{0}\right)g\left(V\right)\right]+g\left(V\right)h\left(T\right)$$

or

$$Z-Z_{0}=g\left(V\right)\left[h\left(T\right)-h\left(T_{0}\right)\right].$$ (21)

Thus, in the case of concurrence, the measured quantity is essentially the product of two functions, the first involving *V* only, and the second only *T.*

We will show in the next section how the concurrence of a family of curves is revealed by the analysis of variance.

## 7. Analysis of Variance

The theoretical basis of the analysis of variance is discussed in reference [1]. The analysis is based on the standard form of the model, as given by eq (13), which can be rewritten as

$$Z_{V,T}=A_{V}+C_{T}+\left(B_{V}-1\right)C_{T}+\epsilon .$$ (22)

To each of the four terms in this expression corresponds a sum of squares, computed as indicated in table 2a.

It is seen from table 2a that the usual interaction term is here partitioned into two parts, (*B_V_*−1)*C_T_* and *ϵ*. Thus, only (*m*−1)(*n*−2) degrees of freedom are available for random error, the remaining (*m*−1) being allocated to the important “slope effect.” The analysis thus provides an answer to the question of how the *m* estimates given by eq (17) are to be pooled: the total number of degrees of freedom for the pooled estimate is (*m*−1)(*n*−2) (rather than *m* (*n*−2), because of the correlation between the *m* separate estimates). The *m*−1 degrees of freedom corresponding to the term (*B_V_*−1)*C_T_* provide a means for testing the “parallelism” of the family of curves. In the case of parallelism the mean square corresponding to the (*B_V_*−1)*C_T_* term will not be significantly larger than the *ϵ* mean square and the model underlying the set of curves becomes the simple additive model of ordinary analysis of variance.

The existence of a point of concurrence is tested by a further partitioning of the interaction sum of squares. The test is based on the theorem proved in the preceding section that a necessary and sufficient condition of concurrence is the existence of an exact linear relation between *f*(*V*) and *g*(*V*). In view of eqs (10a) and (10b), this implies a linear relation between *A_V_* and *B_V_.* But then the correlation between these two quantities is unity. Consequently, the test for concurrence is carried out as follows. First, compute the correlation coefficient $r_{\hat{A},\hat{B}}$ between the quantities ${\hat{A}}_{V}$ and ${\hat{B}}_{V}$. Then partition the (*B_V_*_−1_)*C_T_* term as shown in table 2b. If the mean square for concurrence is significant with respect to that for nonconcurrence *and* the latter is comparable in magnitude to the *ϵ* mean square, there is good evidence that the family of curves pass through a common point. Of course, one can also plot the *m* points $\left({\hat{A}}_{V},{\hat{B}}_{V}\right)$; if an exact straight line (to within *ϵ* error) results, there is concurrence in the family of curves.

## 8. Further Generalization of the Model

Suppose that application of the proposed method to a particular one-parameter family of curves has been unsuccessful. In terms of eq (13), this would be shown by the failure to obtain straight-line relationships when *Z_V, T_* is plotted versus *C_T_*, for particular values of *V*. A natural extension of the procedure is to try a model of the type

$$Z_{V,T}=A_{V}^{\prime }+B_{V}^{\prime }C_{T}+D_{V}C_{T}^{2}$$ (23)

that is, to fit a quadratic, rather than a linear relation, to *Z* as a function of the column averages. If necessary, a polynomial of degree higher than two can be tried. Experience shows that the quadratic model represented by eq (23) may give very satisfactory results where the simpler linear model fails. For computational convenience, it is often advantageous to make the quadratic fit by the method of orthogonal polynomials in *C_T_*, despite the fact that the *C_T_* can, of course, not be expected to be equidistant. The relative advantage of using orthogonal polynomials increases with the number of rows in the table, since all rows are fitted versus a constant set of polynomials in *C_T_.* For the quadratic model, the method of orthogonal polynomials yields the equation

$$Z_{V,T}=A_{V}+B_{V}C_{T}+D_{V}\left[Q\left(C_{T}\right)\right]$$ (24)

where *A_V_* and *B_V_* and *C_T_* are identical with the corresponding quantities used in the linear fit, and *Q*(*C_T_*) is defined by:

$$Q\left(C_{T}\right)=C_{T}^{2}-\left(\frac{\sum_{T}C_{T}^{3}}{\sum_{T}C_{T}^{2}}\right)C_{T}-\left(\frac{\sum_{T}C_{T}^{2}}{n}\right)$$ (25)

where *n* is the number of values of *T* (number of columns). The estimate of *D_V_* is given by

$${\hat{D}}_{V}=\frac{\sum_{T}Z_{V,T}\left[Q\left(C_{T}\right)\right]}{{\sum_{T}\left[Q\left(C_{T}\right)\right]}^{2}}.$$ (26)

The improvement of the quadratic fit over the linear one can be assessed by the corresponding reduction in the sum of squares in the analysis of variance. Denoting the reduction in the sum of squares due to the quadratic term by SS*_D_*, we have:

$${\text{SS}}_{D}=\left(\sum_{V}{\hat{D}}_{V}^{2}\right)\sum_{T}{\left[Q\left(C_{T}\right)\right]}^{2}.$$ (27)

The corresponding number of degrees of freedom is *m*−1, where *m* represents the number of *V* values (number of rows).

## 9. Application to the Compression of Vulcanized Rubber

The data in table 3 are taken from a study of the compression of natural rubber-sulfur vulcanizates [3]. Tabulated are specific volume measurements for pressure values ranging from 1 to 10,000 atm over a temperature range extending from 20 to 80 °C. The analysis was made using the program for the IBM 7090 computer, to be described in the last section. The analysis of variance is shown in table 4. This analysis corresponds to a fit of the data by the empirical formula

$$V=A_{p}+B_{p}C_{T}+\epsilon$$ (28)

where *V* is the measured specific volume, *A_p_* and *B_p_* are two functions of pressure, and *C_T_* is a function of temperature. The symbol *ϵ* represents an “error-term,” including the effect of experimental error as well as that of any inadequacy of eq (28) to represent the data. It is seen that the standard deviation corresponding to this error term is 0.00094. Since the values of specific volume are all of the order of 0.85, the coefficient of variation of the error term is about 0.11 percent.

**Figure 1:**
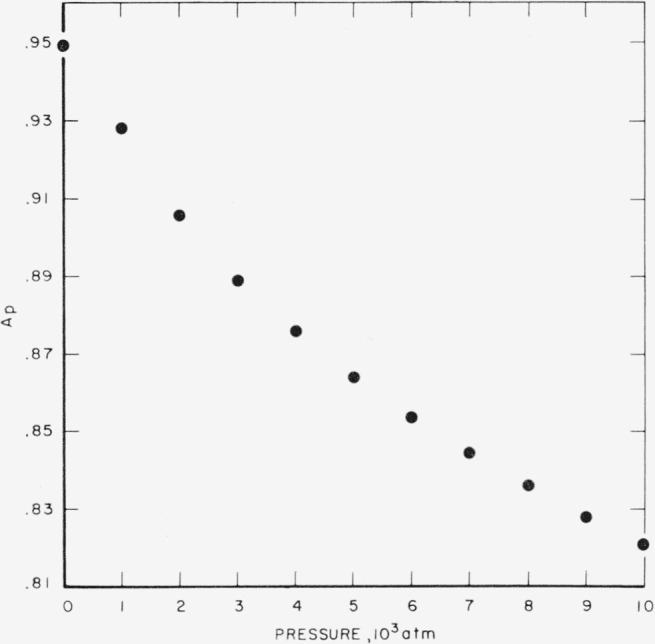
Compression of vulcanized rubber, parameter *A*.

**Figure 2:**
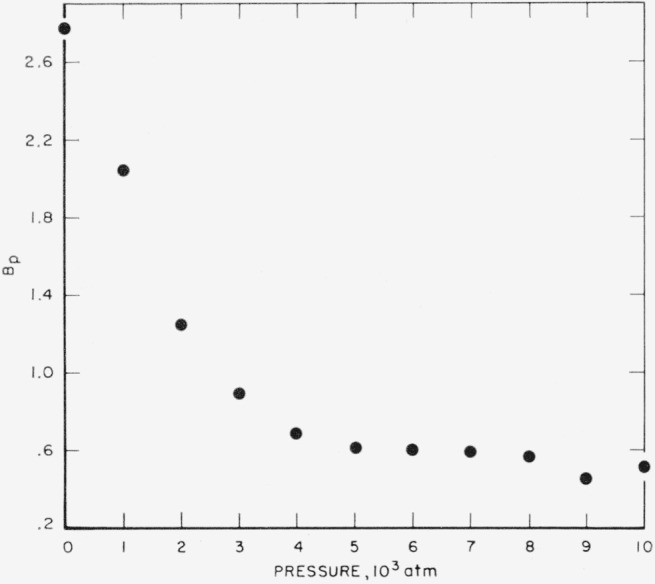
Compression of vulcanized rubber, parameter *B*.

**Figure 3:**
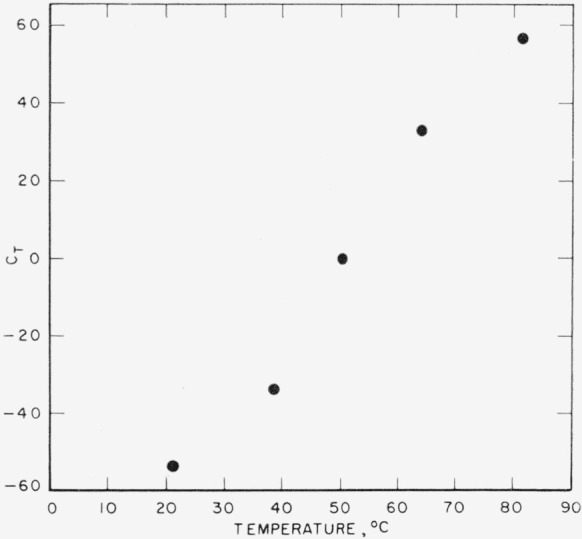
Compression of vulcanized rubber, parameter *C*.

The values of *A_p_, B_p_*, and *C_T_* are listed in table 5. Their relation to pressure and temperature are shown in figures 1, 2, and 3. It is interesting to compare the results of this analysis with those of the conventional analysis of variance for a two-way table. In such an analysis, the effect of “slopes” would not have been separated from that of random interaction. Consequently, the trend shown in figure 2 would have been ignored; i.e., the curve in this figure would have been replaced by a horizontal straight line. The “error-term” would have been inflated by the trend of figure 2 and would have yielded a mean square of 12.37×10^−6^ (the pooled mean square for the last two terms in table 4) corresponding to a standard deviation of error of 0.0035, and a coefficient of variation of roughly 0.4 percent.

By means of figures 1, 2, and 3, the effects of pressure and temperature on specific volume have been quantitatively separated. Figures 1 and 2 represent the effect of pressure; by fitting empirical curves to these graphs, isotherms can be obtained for each of the temperatures included in the study. Figure 3 represents the effect of temperature. It exhibits a possible discontinuity of slope which, if real, would be interpreted as a so-called “glass transition.”

In the next section we will discuss another application, for which an analytic expression will be derived to represent the data.

## 10. Application to the Isotherms of Ethylene

The data for this illustration are taken from a published study of the isotherms of ethylene [2], for temperatures between 0 and 150 °C and pressures up to 3,000 atms. The data for 0 °C were incomplete. A complete rectangular array could be extracted from the data, covering 6 values of temperature (columns), and 40 values of density (rows). However, in order to demonstrate the capabilities of the proposed fitting process, only 13 densities were selected from this set. These data are shown in table 6: they were analyzed by the IBM 7090 program. An examination of the residuals revealed, however, a marked increase in variance with an increase in density. Therefore, the analysis was repeated, after “weighting” the rows, representing densities, by an appropriate factor. This “weighting by rows” is a simple procedure. Let

$$Z\equiv pV=A_{d}+B_{d}C_{T}+\epsilon_{d,T}$$ (29)

and let the variance of *ϵ_d_,_T_* be given by

$$V\left(\epsilon_{d,T}\right)=\frac{K}{\omega_{d}}.$$ (30)

Then, multiplying eq (29) by $\sqrt{\omega_{d}}$ we have:

$$\left(\sqrt{\omega_{d}}\right)\left(Z_{d,T}\right)=\left(\sqrt{\omega_{d}}A_{d}\right)+\left(\sqrt{\omega_{d}}B_{d}\right)C_{T}+\left(\sqrt{\omega_{d}}\epsilon_{d,T}\right).$$

Denoting $\left(\sqrt{\omega_{d}}\right)\left(Z_{d,T}\right)$ by $Z_{d,T}^{*}$ we obtain

$$Z_{d,\;T}^{*}=A_{d}^{*}+B_{d}^{*}C_{T}+\epsilon_{d,\;T}^{*}$$ (31)

where

$$A_{d}^{*}=\sqrt{\omega_{d}}A_{d}$$ (32a)

$$B_{d}^{*}=\sqrt{\omega_{d}}B_{d}$$ (32b)

and

$$V\left(\epsilon_{d,\;T}^{*}\right)=\omega_{d}\frac{K}{\omega_{d}}=K.$$ (33)

Thus, eq (31) now represents a family of curves with constant error-variance; the *C_T_* are redefined in terms of the $Z_{d,\;T}^{*}$ and *A_d_* and *B_d_* are computed from $A_{d}^{*}$ and $B_{d}^{*}$ using eqs (32).

In the present case, the weights *ω_d_* were chosen in accordance with the relation

$$\sqrt{\omega_{d}}=\frac{1}{{\hat{A}}_{d}}$$ (34)

where *A_d_* is of course simply the average of all *pV* values in the row corresponding to density *d.* It follows from this choice and eq (32a), that the estimate of $A_{d}^{*}$ is equal to unity for all values of *d.*

Equation (31) was fitted to the data and gave a coefficient of variation of 0.3 percent. Since the data are believed to have a better precision than is indicated by this coefficient of variation, the fitting process was repeated, using the quadratic model:

$$Z_{d,\;T}^{*}=A_{d}^{*}+B_{d}^{*}C_{T}+D_{d}^{*}\left[Q\left(C_{T}\right)\right]+\epsilon_{d,\;T}^{*}$$ (35)

where *Q*(*C_T_*) is defined by eq (25) and $D_{d}^{*}$ is estimated by a formula similar to eq (26). In terms of the unweighted data, the coefficient of the quadratic term is *D_d_*, where

$$D_{d}^{*}=\sqrt{\omega_{d}}D_{d}.$$ (32c)

The analysis of variance is given in table 7. It should be noted that the latter is in terms of the weighted values, in accordance with eq (35). Thus, the residual variance is a measure of *V*(ϵ^*^), not *V*(*ϵ*). Furthermore, because $A_{d}^{*}=1$ for all *d*, the mean square corresponding to this term is zero. From eqs (30) and (33) we infer that:

$$\sigma_{\epsilon }*=\sqrt{\omega_{d}}\sigma_{\epsilon }$$

which, in view of eq (34) becomes

$$\sigma_{\epsilon }*=\frac{\sigma_{\epsilon }}{{\hat{A}}_{d}}.$$ (36)

Thus, σ_ϵ*_ is roughly equal to the coefficient of variation of *pV.* From the analysis of variance it is seen that this coefficient of variation is equal to approximately 0.3 percent using the simple model of the type of eq (13), and that it is reduced to about 0.07 percent when the more complicated model involving a term in $C_{T}^{2}$ is used. This model can be written

$$Z_{d,\;T}=A_{d}+B_{d}C_{T}+D_{d}\left[Q\left(C_{T}\right)\right]+\epsilon$$

or

$$Z_{d,\;T}=A_{d}^{\prime }+B_{d}^{\prime }C_{T}+D_{d}C_{T}^{2}+\epsilon .$$ (37)

The values of $A_{d}^{\prime }$, $B_{d}^{\prime }$, *D_d_* and *C_T_* resulting from the analysis are given in table 8.

The analysis could be terminated at this point. Using table 8 and eq (37), a value of *Z_d,T_* can be computed for any value of *d* and any value of *T* within the ranges of these variables covered by the data. This can be done by numerical interpolation carried out on the functions $A_{d}^{\prime }$, $B_{d}^{\prime }$, *D_d_*, and *C_T_.*

To obtain a complete empirical representation of the data, one further step is required. The quantities $A_{d}^{\prime }$, $B_{d}^{\prime }$, and *D_d_* must be expressed as calculable functions of the density *d*, and *C_T_* as a calculable function of the temperature *T.* This was done by fitting polynomial expressions to each of these functions, using the data in table 8. In particular, the quantity *C_T_* was satisfactorily fitted by

$$C_{T}=c_{0}+c_{1}T+c_{2}T^{2}.$$

A reduction in the overall number of coefficients is achieved by introducing the quantity

$$C_{T}^{\prime }=\frac{C_{T}-c_{0}}{c_{1}}=T+\frac{c_{2}}{c_{1}}T^{2}.$$ (38)

Then, as can readily be verified, eq (37) can be written in the form

$$Z_{d,\;T}=A_{d}^{\prime \prime }+B_{d}^{\prime \prime }C_{T}^{\prime \prime }+D_{d}^{\prime \prime }{\left(C_{T}^{\prime \prime }\right)}^{2}+\epsilon .$$ (39)

It was found that satisfactory fits were obtained by using a fourth-degree polynomial for *D″* and fifth-degree polynomials for *A″* and *B*″. The coefficients of the fitted polynomials are listed in table 9.

Using these functions, “calculated” values (denoted as ${\hat{Z}}_{d,\;T}$) are obtained for *Z_d, T_* according to the equation

$${\hat{Z}}_{d,\;T}={\hat{A}}_{d}^{\prime \prime }+{\hat{B}}_{d}^{\prime \prime }{\hat{C}}_{T}^{\prime \prime }+{\hat{D}}_{d}^{\prime \prime }{\left({\hat{C}}_{T}^{\prime \prime }\right)}^{2}$$ (40)

in which $\hat{A}\prime \prime$, $\hat{B}\prime \prime$, $\hat{D}\prime \prime$, and $\hat{C}\prime \prime$ are given by the polynomials whose coefficients are listed in table 9. Values of ${\hat{Z}}_{d,\;T}$ for the thirteen densities and six temperatures are given in table 10. A comparison of these values with those of table 6 shows that 90 percent of the fitted values agree with the observed data to within 0.5 percent or better, and that of the remaining ones, all but two agree to within 1 percent. The largest relative deviation is 1.23 percent.

The fitting procedure has therefore been very successful for these data. Since all the data are fitted by a single algebraic expression, interpolation for either pressures or temperatures not used in the fit should be accurate. To test this point, eq (40) was used for interpolation at densities not used in the fitting procedure. It may be recalled that the data used for the fit constituted a selection of 13 densities from a total available set of 40 densities. Values of *pV* were now calculated for all six temperatures and the following additional densities: 111.849, 153.349, 221.48, 245.75, 310.08, 355.43, and 456.85. This last value is outside the range covered by the fit and involves therefore an extrapolation process. The remaining six densities involve only interpolation. Thus, the fitted surface was tested for 42 individual values by interpolation or extrapolation. The results showed that for 35 of these 42 values, the difference “observed minus fitted” was less than 0.5 percent of the observed value. All but three of these differences were smaller than 1 percent of the observed values. The largest difference was equal to 1.21 percent of the observed value. Thus, the values obtained by interpolation are of the same order of precision as those directly fitted. This appears to be generally true for the procedure proposed in this paper, provided that the fits used for the single-variable functions *A, B, C*, and *D* are all of sufficient accuracy.

It is interesting to compare the results of this fit with the equally empirical fitting process used by Michels and Geldermans [2]. These authors fitted each isotherm individually, requiring a total of 42 coefficients for the six isotherms, as contrasted with the 18 coefficients (listed in table 9) required by the present procedure. The residuals obtained by Michels and Geldermans are somewhat smaller than those obtained by the present fit. On the other hand, the procedure used in this paper leads to a single algebraic expression to fit the entire surface. Differentiation is possible both with respect to density and temperature whereas Michels and Geldermans’ fit does not allow for differentiation with respect to temperature.

## 11. Computer Program

A program has been written to fit data to the linear or quadratic models on the IBM 7090 computer. The program was written in Fortran. The original data, fitted parameters, residuals resulting from the fitting procedures, and analysis of variance are printed. Row or column weighting may be used. Provision is also made for transforming the data, for combining rows or columns of the data, for applying specified corrections to individual data, for reversing rows and columns of the data, and for omitting specified rows and columns from the original set of data. The rows and columns of the data are identified by alphabetical or numerical labels so the output is easily interpreted without any coding.

## 12. Further Generalizations

Measurements dependent on two variables are not always given in the form of a complete two-way array, such as table 1. It is often possible, in such cases, to construct such a table by interpolation or curve fitting procedures carried out on subsets of the data within which one of the two variables is held constant.

The presentation in this paper has been in terms of one-parameter families of curves. The method can, however, be used for the analysis of fandlies of curves involving more than one parameter. Applications of this type are now being made.

## 13. Summary

A systematic method has been presented for the empirical fitting of data depending on two variables. Essentially, the method reduces the fitting of surfaces to that of functions of single variables. In the basic model these single-variable functions are completely arbitrary, allowing for great flexibility in applying the method. The adequacy of the model can be evaluated without having to introduce algebraic expressions for the single-variable functions. To obtain a complete algebraic representation of the surface, it is then merely necessary to fit the single-variable functions by any appropriate method.

In certain cases it may be desirable to omit this last step, and still retain a workable model which will express the surface in terms of tabulated functions of single-variables. In that case, numerical interpolation methods must be applied to these tabulated values.

The first example used to illustrate the method deals with the effects of pressure and temperature on the specific volume of certain types of rubber. A quantitative separation of these effects was obtained in terms of tabulated values of three single-variable functions. The fit by means of these functions was within experimental error.

A second example concerned the equation of state of ethylene. The entire set of data was represented by a single algebraic expression and a good fit was obtained. Eighteen coefficients were required by this fit, as against 42 coefficients necessitated by the procedure commonly used for data of this type.

The statistical analysis required for the application of the proposed procedure is presented. In addition to providing estimates for the parameters of the model, the analysis allows for testing the significance of the pertinent effects.

## Figures and Tables

**Table 1:** Schematic of *p-V-T* data

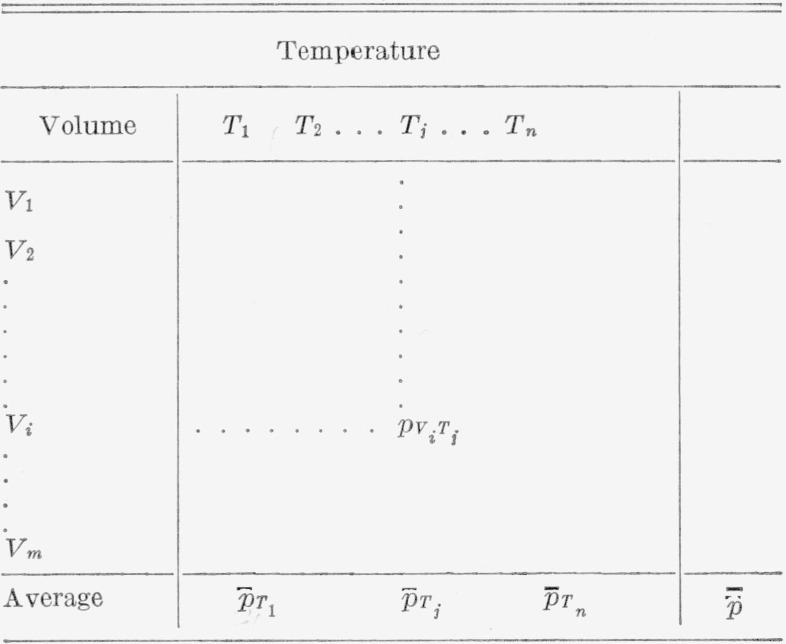

**Table 2a:** Analysis of variance

Term in eq (22)	Degrees of freedom	Sum of squares	Mean square
			
*A_V_*	*m*−1	${\text{SS}}_{A}=n\Sigma_{V}{\hat{A}}^{2}{\;}_{V}$	$\frac{{\text{SS}}_{A}}{m-1}$
*C_T_*	*n*−1	SS*c* = *m*Σ*_T_C*^2^*_T_*	$\frac{{\text{SS}}_{C}}{n-1}$
(*B_V_*−1)*C_T_*	*m*−1	${\text{SS}}_{B\times C}=\Sigma_{V}{\left({\hat{B}}_{V}-1\right)}^{2}\Sigma_{T}C^{2}{\;}_{T}$	$\frac{{\text{SS}}_{B\times C}}{m-1}$
	(*m*−1)(*n*−2)	${\text{SS}}_{\epsilon }=\Sigma_{V}\Sigma_{T}{\left[Z_{V,T}-\left({\hat{A}}_{V}+\hat{B}C_{T}\right)\right]}^{2}$	$\frac{{\text{SS}}_{\epsilon }}{\left(m-1\right)\left(n-2\right)}$

**Table 2b:** Test for concurrence

Term in eq (22)	Degrees of freedom	Sum of squares	Mean square
			
(*B_V_*−1)*C_T_*	*m*−1	SS*_B_*_×_*_C_*	$\frac{{\text{SS}}_{B}{\;}_{\times C}}{m-1}$
Concurrence	1	${\text{SS}}_{\text{conc}}=\left[{\text{SS}}_{B}{\;}_{\times C}\right]\left[r^{2}{\;}_{\hat{A},\;\hat{B}}\right]$	SS_conc_
Nonconcurrence	*m*−2	${\text{SS}}_{\text{nonconc}}=\left[{\text{SS}}_{B}{\;}_{\times C}\right]\left[1-r^{2}{\;}_{\hat{A},\;\hat{B}}\right]$	$\frac{{\text{SS}}_{\text{nonconc}}}{m-2}$

**Table 3:** Specific volume of rubber

Pressure	Temperature, °C				
	21.0	38.5	50.2	64.0	81.5
					
*atm*					
1	0.93397	0.94143	0.94826	0.95639	0.96667
1000	.91678	.92204	.92673	.93344	.94077
2000	.89941	.90189	.90464	.90953	.91360
3000	.88447	.88645	.88880	.89231	.89436
4000	.87214	.87336	.87572	.87864	.87937
5000	.86056	.86188	.86424	.86697	.86680
6000	.85038	.85134	.85410	.85654	.85636
7000	.84113	.84201	.84480	.84717	.84708
8000	.83263	.83345	.83608	.83828	.83828
9000	.82647	.82563	.82859	.82996	.83074
10000	.81834	.81829	.82129	.82249	.82360

**Table 4:** Specific volume of rubber—analysis of variance

Term in eq (28)^a^	Degrees of freedom	Sum of squares	Mean square
			
*A_p_*	10	0.0878718	0.008787
*C_T_*	4	.0009200	.000230
(*B_p_*−1)*C_T_*	10	.0004684	.000047
*ϵ*	30	.0000264	.00000088

a (Equation (28) may be written as follows: $V=A_{p}+C_{T}+\left(B_{p}-1\right)C_{T}+\epsilon .$

**Table 5:** Specific volume of rubber—parameters

Pressure	*A_p_*	*B_p_*	Temperature	*C_T_*
				
1	0.9494	2.7737	21.0	−0.005370
1000	.9280	2.0484	38.5	−.003416
2000	.9058	1.2497	50.2	−.000191
3000	.8893	0.8905	64.0	+.003306
4000	.8758	.6899	81.5	+.005671
5000	.8641	.6149		
6000	.8537	.6020		
7000	.8444	.5986		
8000	.8357	.5658		
9000	.8283	.4576		
10000	.8208	.5167		

**Table 6:** Equation of state for ethylene*a*

Density	Temperature, °C					
	25	50	75	100	125	150
						
19.0407	0.97365	1.07743	1.18010	1.28137	1.38277	1.48309
47.875	.80607	0.92622	1.04361	1.15894	1.27309	1.38615
90.841	.60885	.75053	0.88775	1.02243	1.15528	1.28704
133.083	.47510	.63254	.78765	0.94127	1.09356	1.24486
186.001	.37578	.55506	.73693	.91911	1.10127	1.28330
205.88	.35635	.54767	.74293	.93895	1.13521	1.33117
238.60	.35108	.56984	.79304	1.01706	1.24101	1.46466
266.25	.38459	.63473	.88799	1.14112	1.39357	1.64500
291.80	.46332	.74881	1.03528	1.32004	1.60310	1.88420
315.34	.59374	.91664	1.23807	1.55592	1.87086	2.18230
375.30	1.31315	1.74911	2.17600	2.59290	3.00240	3.40630
415.87	2.2661	2.7896	3.2984	3.7906	4.2745	4.7487
437.03	2.9648	3.5354	4.0890	4.6228	5.1463	5.6596

a The tabulated value is *pV.*

**Table 7:** Equation of state for ethylene—analysis of variance

Term in eq (35)	Degrees of freedom	Sum of squares	Mean square
			
$A_{d}^{*}$	12	0	0
*C_T_*	5	7.53538	1.50708
$\left(B_{d}^{*}-1\right)C_{T}$	12	0.70968	0.05914
$D_{d}^{*}\left[Q\left(C_{T}\right)\right]$	12	.0004263	.0000355
ϵ^*^	36	.0000178	.000000494
			
Residual error, using eq (31)	48	0.0004441	0.00000925

**Table 8:** Equation of state for ethylene—parameters

Density	*A′*	*B′*	*D*	Temperature	*C*
					
19.0407	1.229967	0.559646	−0.177346×10^−2^	25	−0.457050
47.875	1.100321	.636773	−1.353445×10^−2^	50	−.272819
90.841	0.954057	.744060	−2.150476×10^−2^	75	−.089140
133.083	.863068	.845462	−0.591976×10^−2^	100	.092803
186.001	.825883	.998293	2.786081×10^−2^	125	.273447
205.88	.838570	1.072784	3.599158×10^−2^	150	.452757
238.60	.902332	1.225498	3.916304×10^−2^		
266.25	1.011673	1.386588	2.926550×10^−2^		
291.80	1.174651	1.562422	1.179659×10^−2^		
315.34	1.393917	1.746246	−1.032038×10^−2^		
375.30	2.380897	2.298531	−7.853346×10^−2^		
415.87	3.540819	2.724986	−13.286168×10^−2^		
437.03	4.351878	2.957346	−16.109714×10^−2^		

**Table 9:** Equation of state for ethylene coefficients of fitted polynomials^a^

Degree of term in polynomial	*A″*	*B″*	*D″*	*C″*
				
0	1.03565	3.37538×10^−3^	1.75818×10^−6^	0
1	−9.33337×10^−3^	4.59100×10^−5^	−1.07282×10^−7^	1
2	4.43017×10^−5^	−3.05037×10^−7^	1.13865×10^−9^	−1.406662×10^−4^
3	−1.23357×10^−7^	9.77568×10^−10^	−3.71113×10^−12^	
4	8.03331×10^−11^	−2.03756×10^−13^	3.52256×10^−15^	
5	2.71884×10^−13^	−9.87817×10^−16^		

a For *A″, B″*, and *D″* the polynomials are in terms of the density *d*; for *C″*, the polynomial is in terms of the temperature *T.* The equation fitted to the data is *pV* = *A*″+*B″C″+D″*(*C*″)^2^.

**Table 10:** Equation of state for ethylene calculated values

Density	Temperature, °C					
	25	50	75	100	125	150
						
19.0407	0.97650	1.07924	1.18138	1.28291	1.38382	1.48413
47.875	.80066	0.92173	1.04053	1.15710	1.27147	1.38367
90.841	.61040	.75035	0.88793	1.02317	1.15611	1.28676
133.083	.48047	.63589	.79020	0.94342	1.09554	1.24656
186.001	.37525	.55591	.73715	.91892	1.10118	1.28389
205.88	.35326	.54715	.74194	.93757	1.13401	1.33119
238.60	.34675	.56859	.79126	1.01471	1.23889	1.46376
266.25	.38248	.63477	.88716	1.13962	1.39210	1.64457
291.80	.46452	.75090	1.03614	1.32021	1.60311	1.88481
315.34	.59742	.92027	1.24037	1.55772	1.87233	2.18421
375.30	1.31369	1.74885	2.17557	2.59397	3.00415	3.40623
415.87	2.26312	2.78469	3.29348	3.78971	4.27354	4.74519
437.03	2.96876	3.53605	4.08850	4.62635	5.14983	5.65916

## Footnotes

1 Figures in brackets indicate the literature references at the end of this paper.

## References

[1] Mandel John (1961). Non-additivity in two-way analysis of variance. J Am Stat Assoc.

[2] Michels A, Gelderman M (1942). Isotherms of ethylene up to 3000 atmospheres between 0° and 150 °C. Physica.

[b3-jresv67an3p259_a1b] 3Weir, C. E., private communication.

